# Long-term evaluation of the COVID-19 pandemic impact on acute stroke management: an analysis of the 21-month data from a medical facility in Tokyo

**DOI:** 10.1007/s13760-022-01979-0

**Published:** 2022-05-26

**Authors:** Takashi Mitsuhashi, Joji Tokugawa, Hitoshi Mitsuhashi

**Affiliations:** 1 Department of Neurosurgery, Juntendo University Nerima Hospital, Takanodai 3-1-10, Nerima, Tokyo, 177-8521 Japan; 2 School of Commerce, Waseda University, Nishi-Waseda 1-6-1, Shinjuku, Tokyo, 169-8050 Japan

**Keywords:** COVID-19, Pandemic impacts, Stroke care pathways, Admissions, Time measures

## Abstract

**Introduction:**

The coronavirus disease 2019 (COVID-19) pandemic has caused a global public health crisis and profoundly impacted acute treatment delivery. This study conducted long-term evaluations of the impact of the pandemic on acute stroke management.

**Methods:**

Data from a university-owned medical facility in Tokyo, Japan, were retrospectively analyzed. The number of hospital admissions for stroke and time metrics in the management of patients with acute ischemic stroke were evaluated. A year-over-year comparison was conducted using data from April 2019 to December 2021 to assess the impact of the pandemic.

**Results:**

The year-over-year comparison demonstrated that the number of admissions of patients with stroke and patients who underwent magnetic resonance imaging (MRI), intravenous recombinant tissue plasminogen activator (rt-PA), and thrombectomy during the pandemic remained comparable to the pre-COVID data. However, we found a decrease in the number of admissions of patients with stroke alerts and stroke when hospital cluster infection occurred at this facility and when the region hosted the Tokyo Olympics games during the surge of infection. The door-to-computed tomography time in 2021 was affected. This is plausibly due to the reorganization of in-hospital stroke care pathways after hospital cluster infection. However, no significant difference was observed in the onset-to-door, door-to-MRI, door-to-needle, or door-to-groin puncture times.

**Conclusions:**

We did not observe long-term detrimental effects of the pandemic at this site. Prevention of hospital cluster infections remains critical to provide safe and timely acute stroke management during the pandemic.

## Introduction

An acute ischemic stroke is a critical event. For early restoration of blood flow in acute ischemic stroke, arterial recanalization must be provided by administering recombinant tissue plasminogen activator (rt-PA) or performing thrombectomy in a timely and safe manner. As the coronavirus disease 2019 (COVID-19) pandemic requires restrictive contact precautions and reorganizations of established stroke care pathways, it is important to evaluate the pandemic impact on acute stroke management and present findings to healthcare providers and policymakers to optimize pre- and in-hospital workflows.

Table 1 summarizes 32 papers on the global pandemic impacts published in 2020 and 2021. These studies evaluated the number of stroke-related admissions and key time process measures such as onset-to-door time, door-to-computed tomography (CT) time, and door-to-groin puncture time because delays in these measures limit the restoration of perfusion in acute ischemic stroke.

**Table 1:** Previous studies on the impacts of the pandemic in acute stroke management

	Data	Admissions	Onset-to-door	Door-to-CT	Door-to-groin puncture
Agarwal et al. [1]	March 1 to May 15, 2020, New York	↓	→	↑	↑
Amukoutuwa et al. 2]	March 1 to May 10, 2020, Australia	↓	n.a	n.a	n.a
Brunetti et al. [3]	March 11 to May 4, 2020, Rome Italy	↓	↑	n.a	↑
D'Anna et al. [4]	March 23 to June 30, 2020, London	↓	↑	→	→
Drenck et al. [5]	March 13, 2020 to February 28, 2021, Denmark	↓	n.a	n.a	n.a
Frisullo et al. [6]	March 11 to April 11, Rome, Italy	↓	↑	→	↑
Fuentes et al. [7]	February 25 to April 25, 2020, Madrid, Spain	↓	n.a	n.a	↑
Ghoreishi et al. [8]	February 18 to July 18, 2020, Zanjan Province, Iran	↓	n.a	n.a	n.a
Jasne et al. [9]	January to April, 2020, New Haven, Connecticut	↓	→	n.a	→
Kansagra et al. [10]	February to April, 2020, US	↓	n.a	n.a	n.a
Katsanos et al. [11]	March 1 to April 30, 2020, Ontario, Canada	n.a	→	↑	→
Kim et al. [12]	March 1, 2020 to February 28, 2021, Busan, Korea	↓	n.a	n.a	n.a
Koge et al. [13]	April to July, 2020, Japan	↓	→	↑	↑
Kristoffersen et al. [14]	January to September, 2020, Norway	↓	n.a	n.a	n.a
Kwan et al. [15]	January to April, 2020, UK	↓	→	→	→
Lee et al. [16]	February 18 to April 17, 2020, Daegu, Korea	n.a	↑	→	→
Nogueira et al. [17]	March 1 to May 31, 2020, 40 countries	↓	n.a	n.a	n.a
Nogueira et al. [18]	March 1 to June 30, 2020, 70 countries	↓	n.a	n.a	n.a
Padmanabhan et al. [19]	March 15 to April 14, 2020, UK	↓	→	n.a	→
Raymaekers et al. [20]	March to May, 2020, Belgium	↓	n.a	n.a	→
Richter et al. [21]	March 16 to May 15, 2020, Germany	↓	n.a	n.a	n.a
Rudilosso et al. [22]	March 1 to 31, 2020, Barcelona	↓	→	→	→
Sharma et al. [23]	March 23 to April 19, 2020, Boston	↓	→	n.a	n.a
Siegler et al. [24]	March to July, 2020, US	n.a	n.a	↓	→
Teo et al. [25]	January 23 to March 25, 2020, Hong Kong	↓	→	n.a	→
Tiedt et al. [26]	March to May 2020, Germany	→	→	→	↑
Uchino et al. [27]	March 9 to April 2, 2020, Ohio	↓	→	→	→
Velez et al. [28]	March 11 to April 2020, Chicago	↓	↑	n.a	n.a
Velilla-Alonso et al. [29]	March 14 to May 14, 2020, Spain	↓	↑	→	→
Vollmuth et al. (2021)	March to June, 2020, Germany	↓	n.a	n.a	n.a
Wong et al. [30]	April, 2020 to January, 2021, North Carolina	↓	n.a	n.a	n.a
Wu et al. [31]	January 24 to April 29, 2020, Beijing	↓	→	n.a	→

↓: decreased, ↑ increased, → did not change

Although most studies agreed upon a decline in the number of admissions during the pandemic, their findings regarding key process time measures are inconclusive. A potential reason for this inconclusiveness might be variations in healthcare systems across different countries and regions. Another reason might be the use of short-term data, with some exceptions [5, 30], which might capture immediate responsive effects shortly after the beginning of the pandemic [3, 31].

Therefore, this study aimed to analyze the pandemic’s long-term effects by comparing the data after the beginning of the COVID-19 pandemic regarding acute treatment delivery for patients with stroke with the pre-COVID data in 2019.

## Methods

### Data site

The data used in this study are available from the corresponding author upon reasonable request.

This was a retrospective single-center observational study at a medium-sized facility owned by a medical university hospital in Tokyo, Japan. This is the only primary 24/7 medical center in the locality. We used all the data of the patients with stroke alerts admitted to this facility.

In 2020 and 2021, metropolitan Tokyo experienced four waves of the COVID-19 pandemic. During the pandemic, the Japanese government issued four country-wide stay-at-home orders: (1) April 7–May 25, 2020; (2) January 7–March 21, 2021; (3) April 25–June 20, 2021; and (4) July 12–September 30, 2021. We used the 21-month data from April 1, 2020, to December 31, 2021, as the pandemic period data and from January 1, 2019, to December 31, 2019, as the baseline data for making year-over-year comparisons.

Since the pandemic, this facility has optimized stroke care pathways to protect frontline healthcare workers against infections. In April 2020, it implemented standard precautions, required the workers to use protective equipment, set up multiple hygienic barriers outside the facility to triage transported patients with COVID-like symptoms, and made a dedicated pathway for patients “suspected” to have COVID-19. Moreover, in August 2020, the facility implemented decontamination procedures in a depressurized room. Despite these efforts, hospital cluster infection occurred in September 2020, whereby the facility closed emergency admission from September 30 to October 17, 2020. After the reopening, all the transported patients were required to undergo triage in the clean booths outside.

## Measurements

This study comprised two analyses. In the first analysis, this study compared the number of stroke-related admissions and acute treatments from April 2020 to December 2021 with the number in the same period in 2019. We evaluated the number of patients admitted to the facility with stroke alerts, patients diagnosed with a stroke, and patients who underwent magnetic resonance (MR) imaging, rt-PA, and thrombectomy.

In the second analysis, this study used key process time measures for stroke care, including time intervals from (1) symptom onset-to-door, (2) door-to-CT, (3) door-to-MR imaging, (4) door-to-needle, and (5) door-to-groin puncture. These measures indicate the time frame for acute stroke treatment. Onset-to-door time represents the time interval from stroke onset to hospital admission. Door-to-CT and door-to-MR imaging represent time intervals from hospital admission to the first two phases of in-hospital care pathways. At this facility, after initial triage and examination, a CT scan is first performed for patients with suspected acute stroke, followed by MR imaging if CT images show no hemorrhagic lesion. The physicians perform thrombolysis and thrombectomy in patients with ischemic lesions, with or without large vessel occlusion on MR imaging. The facility’s emergency room and CT room co-locate with the emergency entrance on the first floor. The distances to the emergency room and CT room were 10 and 15 m, respectively. The MR imaging room was located adjacent to the CT room. We also evaluated the door-to-needle time (the time interval from hospital admission to the initiation of recombinant tissue plasminogen activator (rt-PA) drug treatment) and the door-to-groin puncture time of patients who underwent thrombectomy.

## Statistical analysis

In the first analysis, we assessed the pandemic effects by reporting the monthly averages of the aforementioned numbers in 2019, 2020 (from April to December), and 2021. We used the 2019 data as the baseline pre-COVID data and compared them with those of the 2020 and 2021 data as the pandemic data. We performed Mann–Whitney non-parametric *U* tests (MW tests) to assess statistical differences. As all patients admitted to this facility with stroke alerts underwent CT scans, we did not report the door-to-CT statistics. Additionally, to capture potential seasonal fluctuations in patient volumes during the pandemic, we reported the number of transported patients and patients with stroke per day in each month and conducted t-tests and MW tests.

In the second analysis, we reported the results of *t*-tests and MW tests and reported whether the means of the time measures during the treatment periods were significantly different from those of the control periods.

## Results

### Analysis 1: volume

In 2019, the monthly average of patients presenting with signs of a stroke or transient ischemic attack (TIA) was 29.75 patients (Table 2). We observed a decrease in this number in both 2020 (23.33 patients, MW statistics = 80.5, *p* = 0.064) and 2021 (27.00 patients, MW statistics = 89.5, *p* = 0.325), but these differences from the baseline pre-COVID data were not statistically significant at the *α* = 0.05 level. We also found a decrease in the monthly average number of patients treated with rt-PA from 3.83 in 2019 to 2.67 in 2020 (MW statistics = 59.5, *p* = 0.385) and 2.25 in 2021 (MW statistics = 93.5, *p* = 0.090), but these results were not statistically significant at the *α* = 0.05 level. The number of patients who were diagnosed with stroke and underwent MRI and thrombectomy remained constant throughout the study period (Table 2).

**Table 2:** Monthly average number of patients and stroke care treatments

	All patients	Stroke patients	MRI	rt-PA	Thrombectomy
2019, 1/1 to 12/31	29.75	22.75	19.17	3.83	1.42
2020, 4/1 to 12/31	23.33	17.78	16.67	2.67	1.56
MW	80.5	75.0	69.5	59.5	16.0
*p*	0.063	0.143	0.284	0.385	0.405
2021, 1/1 to 12/31	27.00	20.17	19.50	2.25	1.75
MW	89.5	88.0	72.5	93.5	36.0
*p*	0.325	0.368	1.000	0.090	0.711

*MW* Mann–Whitney non-parametric *U* test statistics

**Fig. 1:**
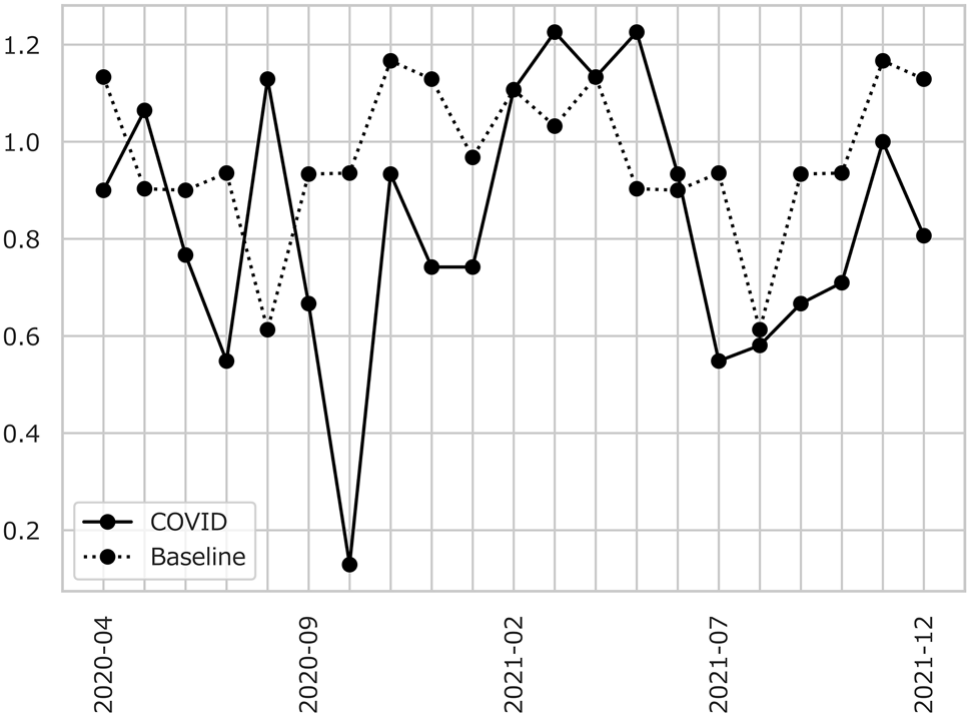
Patients with stroke alerts per day. This figure shows changes in the monthly average of the number of patients with stroke alerts per day. The solid line indicates the pandemic data. The observation periods during the pandemic started in April 2020 and ended in December 2021. The dotted line represents the baseline data in 2019. The number of patients dropped sharply in October 2020 due to hospital cluster infection

To further understand the significant decline in the number of patients with stroke alerts, we made month-to-month comparisons between the pre-COVID and COVID periods (Table 3 and Fig. 1). The daily average number of patients admitted to the facility decreased from 0.94 in July 2019 to 0.55 in July 2020 (t-statistics = 2.249, *p* = 0.029, MW statistics = 602.0, *p* = 0.059); however, the number of patients with stroke did not decrease statistically (*t*-statistics = 1.447, *p* = 0.154, MW statistics = 555.5, *p* = 0.232), suggesting a decrease in the admissions of stroke mimics only.

**Table 3:** The number of patients per day

	All patients						Stroke patients					
	*N*	*N* in 2019	*t*-stats	*p*	MW	*p*	*N*	*N* in 2019	*t*-stats	*p*	MW	*p*
2020-04	0.90	1.13	1.013	0.316	495.5	0.480	0.73	0.73	0.000	1.000	427.0	0.718
2020-05	1.06	0.90	0.643	0.523	437.5	0.526	0.77	0.77	0.000	1.000	499.5	0.777
2020-06	0.77	0.90	0.592	0.556	501.0	0.424	0.73	0.73	0.000	1.000	467.0	0.790
2020-07	0.55	0.94	2.249	0.029	602.0	0.059	0.42	0.65	1.447	0.154	555.5	0.232
2020-08	1.13	0.61	2.300	0.025	329.5	0.024	0.87	0.55	1.533	0.131	389.5	0.164
2020-09	0.67	0.93	1.267	0.211	507.0	0.363	0.53	0.63	0.574	0.568	478.0	0.650
2020-10	0.13	0.94	4.702	0.000	742.5	0.000	0.03	0.68	4.671	0.000	730.0	0.000
2020-11	0.93	1.17	0.903	0.370	514.0	0.324	0.63	1.07	1.793	0.078	575.0	0.048
2020-12	0.74	1.13	1.646	0.105	573.5	0.167	0.52	0.84	1.597	0.116	570.5	0.167
2021-01	0.74	0.97	0.830	0.410	566.0	0.195	0.55	0.74	0.894	0.375	557.5	0.229
2021-02	1.11	1.11	0.000	1.000	388.5	0.959	0.93	0.93	0.000	1.000	392.0	1.000
2021-03	1.23	1.03	-0.747	0.458	417.0	0.340	0.90	0.68	-1.125	0.265	375.5	0.108
2021-04	1.13	1.13	0.000	1.000	444.0	0.930	0.80	0.73	-0.325	0.747	404.0	0.468
2021-05	1.23	0.90	-1.048	0.300	445.5	0.608	0.90	0.77	-0.499	0.620	470.0	0.879
2021-06	0.93	0.90	-0.151	0.881	447.5	0.975	0.63	0.73	0.531	0.597	474.0	0.704
2021-07	0.55	0.94	1.980	0.052	610.0	0.049	0.35	0.65	1.800	0.077	591.0	0.076
2021-08	0.58	0.61	0.177	0.860	468.5	0.857	0.35	0.55	1.186	0.241	540.5	0.326
2021-09	0.67	0.93	1.153	0.254	524.0	0.241	0.63	0.63	0.000	1.000	466.5	0.793
2021-10	0.71	0.94	1.113	0.270	538.5	0.382	0.48	0.68	1.068	0.290	552.0	0.263
2021-11	1.00	1.17	0.681	0.499	495.0	0.490	0.73	1.07	1.522	0.134	535.0	0.184
2021-12	0.81	1.13	1.267	0.210	566.0	0.206	0.71	0.84	0.603	0.549	512.0	0.638

The table compares the daily average number of transported patients and patients with stroke for each month during the coronavirus disease (COVID) periods with the corresponding monthly pre-COVID data. *t*-stats means *t* statistics of two-group comparisons of means (two-sided)

*t-stats* *t* statistics of two group mean comparison (two-sided), *MW* Mann–Whitney non-parametric *U* test statistics

Substantial drops were observed in October 2020 (Table 3 and Fig. 1). The number of patients admitted and patients with stroke dropped from 0.94 in 2019 to 0.13 in 2020 and from 0.68 in 2019 to 0.03 in 2020, respectively. We also found a systematic decline in July 2021. The number of patients dropped from 0.94 in 2019 to 0.55 in 2021 (*t*-statistics = 1.980, *p* = 0.052, MW statistics = 610.0, *p* = 0.049), while that of patients with stroke decreased from 0.65 in 2019 to 0.35 in 2021 (*t*-statistics = 1.800, *p* = 0.077, MW statistics = 591.0, *p* = 0.076).

### Analysis 2: key process time measures

**Fig. 2:**
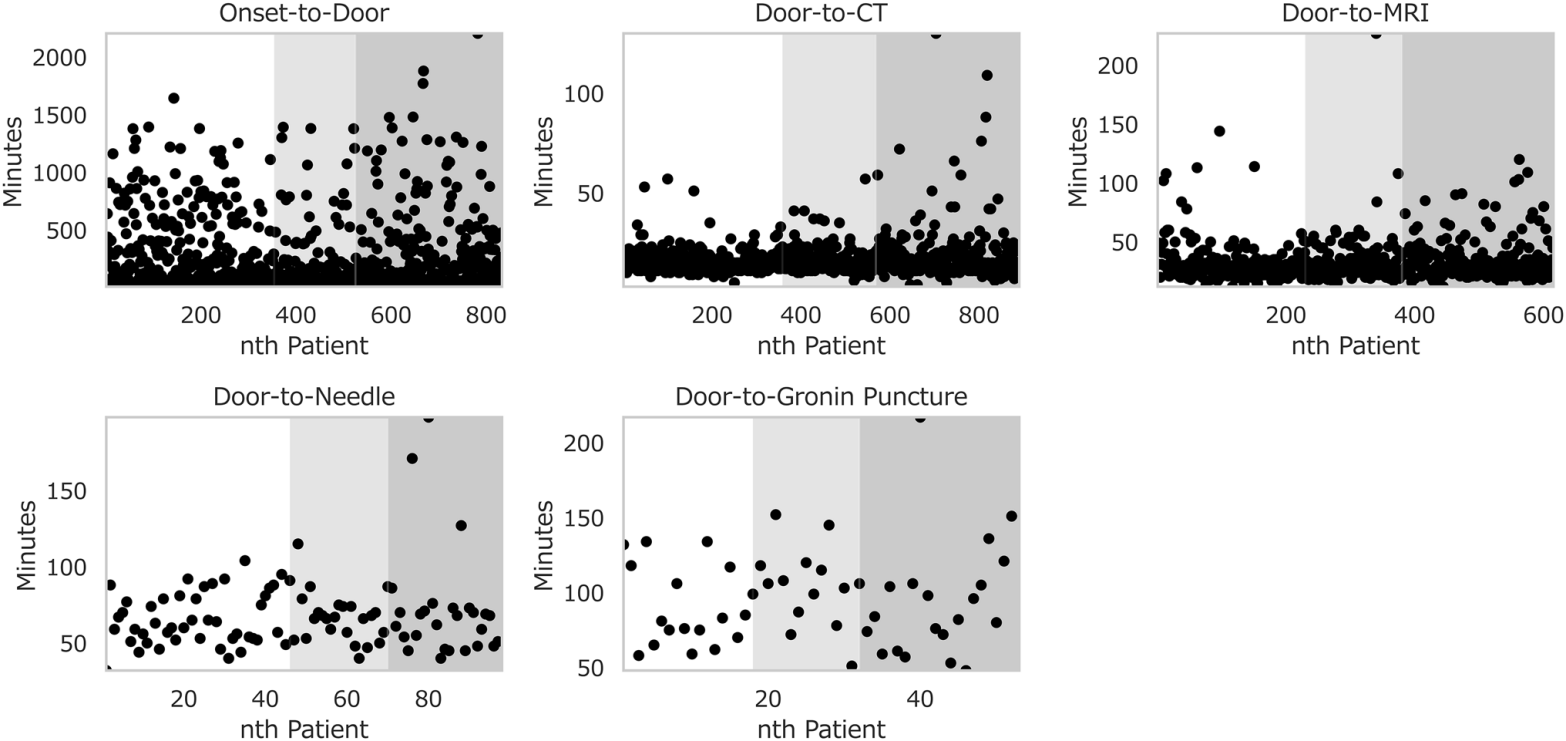
Key process time measures. The figures show the five key process time measures of each patient in minutes. The white areas represent the baseline data in 2019, whereas the lighter and darker grey areas indicate the pandemic data in 2020 (April to December) and 2021, respectively. As not all patients admitted to the facility received the same treatment, the numbers of patients included in each of these panels are different

We evaluated the pandemic impact on five key process time measures (Fig. 2 and Table 4). The only time measure that worsened was the door-to-CT time in 2021. In 2019, the door-to-CT time was 15.19 min with a standard deviation of 5.56 min, whereas, in 2021, it was 17.55 min with a standard deviation of 12.91 min. The mean difference, 2.36 min, was statistically significant (*t*-statistics = − 3.030, *p* = 0.003, MW statistics = 54,544.0, *p* = 0.249). However, we found no significant difference in the door-to-MR imaging, door-to-needle, and door-to-groin puncture times, suggesting that the overall quality of acute stroke care measured with time intervals did not decrease during the pandemic.

**Table 4:** Effects on the key process time measures

	2019, 1/1 to 12/31			2020, 4/1 to 12/31							2021, 1/1 to 12/31						
Time interval	*N*	Mean	SD	*N*	Mean	SD	*t*-test	*p*	MW	*p*	*N*	Mean	SD	*t*-test	*p*	MW	*p*
Onset-to-Door	329	311.56	330.33	189	193.05	283.90	4.304	0.000	39,870.5	0.000	308	276.63	353.20	1.287	0.199	54,815.5	0.074
Door-to-CT	357	15.19	5.56	204	15.99	6.31	1.507	0.133	33,697.5	0.140	322	17.55	12.91	-3.030	0.003	54,544.0	0.249
Door-to-MRI	230	31.89	16.57	147	32.69	20.60	-0.397	0.691	16,592.5	0.762	234	34.11	17.39	-1.405	0.161	24,852.0	0.154
Door-to-needle	46	65.98	17.40	24	66.46	16.00	-0.116	0.908	539.5	0.882	27	72.15	36.93	-0.817	0.420	646.0	0.779
Door-to-groin puncture	17	90.00	27.39	14	103.79	26.93	-1.408	0.170	84.0	0.171	21	94.57	39.06	-0.423	0.675	177.0	0.977

*SD* standard deviations, *t-stats* t statistics of two group mean comparison (two-sided), *MW* Mann–Whitney non-parametric U test statistics

## Discussion

The COVID-19 pandemic has threatened global and national healthcare systems. As the pandemic gave rise to the need for reorganization of pre- and in-hospital stroke care pathways, one of the threats that previous studies [1, 6] reported is the reduced quality of acute stroke management. Previous studies evaluated pre- and in-hospital performance indicators such as the number of patients admitted who presented with signs of stroke or TIA and door-to-groin puncture time.

The findings in these studies are rather mixed, with some reporting detrimental impacts [28, 29], whereas others report limited impacts [26]. One potential source of such disagreements is the duration of the observation periods. The mean of the observation periods in 32 papers was 3.63 months with a standard deviation of 2.94 months (Table 1). Drenck et al. [5] and Kim et al. [12] used the longest observation period data of 12 months. In this study, we used the 21-month pandemic data at a medical facility in Tokyo, Japan, evaluated the long-term pandemic impacts, and conducted a retrospective single-center observational study.

In our first analysis, we studied the effects of the monthly average number of patients and stroke care treatments. We demonstrated a significant decline in the number of admissions of patients with stroke alerts in 2020. In October 2020, the daily average number of patients with stroke alerts and patients with stroke dropped by 42% and 36%, respectively. We attribute the decline in 2020 to the hospital cluster infection in October 2020, which caused the facility to close emergency admission from September 30 to October 17, 2020.

Another systematic decline occurred in July 2021. In addition to the surge of infections from 12,977 in June 2021 to 44,448 in July 2021 (342% increase), the region hosted the Olympic games in that month. Games during the pandemic sparked intense public debates. There is a possibility that social anxiety might raise patients’ fear of COVID-19, which might cause delays in seeking help. This finding suggests that healthcare providers and policymakers should evaluate the value of hosting large social events such as the Olympic games with a consideration of this indirect effect.

Despite these significant differences, we did not observe any systematic differences in other periods between the pre-COVID and COVID periods, leading us to conclude that COVID-19 did not have any substantial impacts on the number of hospital admissions as well as that of stroke care treatments. In our analysis using daily average data, we found no initial decline even shortly after the beginning of the pandemic in April 2020. This might be due to the limited magnitude of the pandemic and the resulting low social fear of in-hospital infections in this region. The highest daily number of COVID-19 cases in metropolitan Tokyo during the observation period was 5908 (August 13, 2021), whereas that in New York State and California was 85,476 (December 31, 2021) and 50,913 (December 31, 2021), respectively.

Our interpretation based on the low social fears of in-hospital infections is in line with our findings in the second analysis (Table 4). The onset-to-door time would significantly increase if patients developed fear; however, we observed that it decreased in 2020 and 2021. The decline in the onset-to-door time suggests that the decrease in the number of patients treated with rt-PA in 2021 (Table 2) did not result from delays in the onset-to-door time.

In the second analysis, we studied the effects of other key process time measures. Almost all the measures remained constant throughout the study period, suggesting that the facility managed to avoid any hazardous delays in in-hospital stroke care that COVID-19 could cause. We did not find substantial increases in the onset-to-door, door-to-MR imaging, door-to-needle, or door-to-groin puncture times. Our results are consistent with other studies that did not report such a delay [15, 22]. However, we observed significant delays in the door-to-CT time in 2021, which is plausibly due to the facility’s optimization of the stroke care pathways after hospital cluster infection in October 2021. The renewed protocols enhanced the protection of frontline healthcare workers against infections but could have increased the mean door-to-CT time in 2021.

An implication of our study is the importance of preventing hospital cluster infections. The cluster infection not only reduced the number of admissions but also required additional reorganization in stroke care pathways. As a result of the hospital cluster infection, the facility made responsive and reactive actions, requiring longer adaptations and learning than preventive actions. The pandemic impacts could be reduced further if medical facilities take preemptive rather than remedial actions. Healthcare providers and policymakers should encourage medical facilities to allocate more resources to prevent hospital cluster infections.

Our study had several limitations. First, this study was based on data collected from a single medical facility in a specific area. Further research using long-term data from other countries and regions is needed to enhance generalizability. Second, although the number of stroke admissions and the time metrics are important to assess the pandemic effects, this study did not examine the quality of treatment received by patients and their overall health and welfare. The goal of acute stroke management is to decrease morbidity and mortality [8, 9]. Future research should focus on these aspects using long-term data. Third, our findings suggest some burden on medical workers because of the pandemic. Future research needs to capture how the pandemic impacts not only patients but also medical workers.

## Conclusions

We conclude that the number of patient admissions and stroke care treatments, as well as key time process measures, were not affected during the COVID-19 pandemic. However, preventing hospital cluster infections remain critical to provide safe and timely acute stroke management during the pandemic.

## Data Availability

The data used in this study are available from the corresponding author upon reasonable request.

## Abbreviations

COVID-19: Coronavirus disease 2019
MRI: Magnetic resonance imaging
CT: Computerized tomography
rt-PA: Intravenous recombinant tissue plasminogen activator
MW: Mann–Whitney non-parametric U tests
